# Low Latency and High Data Rate (LLHD) Scheduler: A Multipath TCP Scheduler for Dynamic and Heterogeneous Networks

**DOI:** 10.3390/s22249869

**Published:** 2022-12-15

**Authors:** Tabassum Lubna, Imtiaz Mahmud, You-Ze Cho

**Affiliations:** 1 School of Electronic and Electrical Engineering, Kyungpook National University, Daegu 41566, Republic of Korea; 2 Lawrence Berkeley National Laboratory, Berkeley, CA 94720, USA

**Keywords:** data rate, delay, MPTCP, schedulers

## Abstract

The scheduler is a crucial component of the multipath transmission control protocol (MPTCP) that dictates the path that a data packet takes. Schedulers are in charge of delivering data packets in the right order to prevent delays caused by head-of-line blocking. The modern Internet is a complicated network whose characteristics change in real-time. MPTCP schedulers are supposed to understand the real-time properties of the underlying network, such as latency, path loss, and capacity, in order to make appropriate scheduling decisions. However, the present scheduler does not take into account all of these characteristics together, resulting in lower performance. We present the low latency and high data rate (LLHD) scheduler, which successfully makes scheduling decisions based on real-time information on latency, path loss, and capacity, and achieves around 25% higher throughput and 45% lower data transmission delay than Linux’s default MPTCP scheduler.

## 1. Introduction

Modern devices possessing various communication interfaces are becoming ubiquitous due to the rapid growth of advanced technologies. The simultaneous usage of several communication interfaces is projected to deliver faster throughput and decrease data transmission latency [1]. Regarding dependable data delivery by using concurrent communication over multiple communication interfaces, the Internet engineering task force (IETF) has introduced a multipath transmission control protocol (MPTCP) as the transport layer protocol. The MPTCP is an extension of the transmission control protocol (TCP), which is a widely accepted transport layer protocol for reliable data transfer [2].

The MPTCP includes three fundamental tools for controlling data transmission: a path manager, a congestion control algorithm (CCA), and a scheduler [3]. The path manager is in charge of establishing end-to-end paths between the sender and the receiver. Depending on the status of the route, it might add or remove it. Each route between the sender and recipient is considered a subflow (SF). The CCA determines the congestion window (CWND), which is the amount of data that may be sent via each of the SFs. The CCA seeks to make maximum use of the underlying network while equitably sharing available resources with other flows in that network. Schedulers decide which SF a data packet should traverse. Its purpose is to prevent head-of-line (HoL) blockage at the receiver by ensuring orderly packet delivery. This study aims to improve the performance of the MPTCP schedulers.

Today’s Internet is a highly complex network integrating wired and wireless links where the traffic load fluctuates rapidly in real-time. Moreover, events like handoffs, decline in signal strength, and user movement typically produce considerable packet losses in mobile networks [4]. The 4G/5G and WiFi networks are the two main communication interfaces that current devices use. The properties of each of these networks vary, particularly in terms of latency, bandwidth (BW), and path loss. The task of MPTCP schedulers is quite challenging due to the various characteristics of these networks. Often the throughput of the MPTCP connection declines, owing to HoL blockage induced by out-of-order delivery [5]. To solve this complex problem, numerous research studies have presented a variety of schedulers [6,7,8]. However, these schedulers largely consider the latency as the core of the scheduling algorithm, disregarding the other crucial metrics, such as BW and path losses. Particularly in wireless networks, where route factors, such as packet loss rate and BW change fast in real-time, it is critical that the scheduler consider these parameters in order to make the best scheduling decision. Otherwise, making a scheduling decision without considering the route characteristics may result in increased packet losses and transmission delay, culminating in complicated HoL blocking at the receiver, extending the delay even more. The end result might be even worse performance than the single path TCP [9,10,11]. Therefore, an intelligent scheduler that takes into account path characteristics, such as path loss and BW, as well as other relevant information, is necessary to make the best scheduling decision.

The present MPTCP stack includes the knowledge of both BW and path losses by continually monitoring the data-rate of each path in real-time [12]. Due to the fact that it provides a thorough picture of the continually changing network in real-time, this information may be crucial for developing the scheduling algorithm. As a result, we suggest a new scheduler, called the low latency and high data rate (LLHD), based on the information that is available. The following are the main contributions:

- In this work, we offer LLHD, a unique MPTCP scheduler that, in addition to taking network characteristics like latency into account, also takes BW and packet losses into account in order to make an effective scheduling decision in real-time;
- LLHD is able to outperform schedulers used currently by achieving a better throughput with less latency for data transmission;
- Unlike other schedulers, LLHD can respond to changes in networks that fluctuate dynamically and deliver the highest throughput with the shortest data transmission time.

The remainder of the paper is structured as follows: a summary of the existing MPTCP schedulers is provided in Section 2, followed by a detailed description of the proposed LLHD in Section 3, an evaluation of the performance of each scheduler under consideration in Section 4, and a conclusion in Section 5.

## 2. Related Works

This section briefly describes the existing schedulers available in the MPTCP Linux Kernel [9]. However, as mentioned earlier, none of them considers all the available network information and fails to produce the expected results in changing network scenarios.

### 2.1. Shortest RTT Scheduler

Quentin et al. introduced the shortest RTT (SRTT) scheduler. It is the default scheduler in the MPTCP Linux Kernel. The packets are allocated to the SF, which has the least RTT among the available SFs and has space in its CWND. When the CWND of the SF having the minimum RTT is full, only then is the data sent through the SF having the next minimum RTT, based on the availability of space in its CWND. However, once the CWND of the shortest RTT subflow becomes available, data transmission is resumed through that subflow. This strategy simply concentrates on transferring the packets in the fastest reasonable period [13].

### 2.2. Round-Robin

In the round-robin (RR) scheduler, the SF for sending a packet is decided in a round-robin fashion. It circularly chooses one SF from all the SFs with available CWND. This scheduling neither focuses on short-time delivery, nor aims to eliminate HoL blockage [14].

### 2.3. Redundant

This scheduler sends the same data through all SFs that have space in CWND. This approach of broadcasting the same data over numerous communication interfaces helps lower the HoL blocking at the receiver, hence providing short data delivery latency. However, conveying the same packet wastes significant BW, which may be utilized for delivering other vital data by developing an effective scheduling system [15].

### 2.4. Earliest Completion First

The earliest completion first (ECF) scheduler aims to create an intelligent scheduling option by considering the RTT of the SFs, their available CWND, and the amount of data to be delivered. In an abstract form, in determining the scheduling choice, it tries to anticipate the arrival time of the packets at the receiver. Then it schedules the packets through the available SFs in such a way that the data arrives in line at the receiver [16]. Thus, it tries to fully utilize all the resources, while at the same time obtain better throughput and lower delay. However, in the modern Internet, wireless links are fairly common. The wireless links’ characteristics change in real-time with the mobility of the user and ECF is caught off guard in such scenarios, as it does not consider packet losses or real-time changes in the path characteristics. Therefore, it cannot always perform well and cannot be applied as a generic scheduler [17].

### 2.5. Block Estimation

The block estimate (BLEST) scheduler provides a way to anticipate HoL blocking. Depending on the RTT and send window of an SF, it assesses whether or not transmitting data over that SF could induce HoL blocking at the receiver. If it identifies such a possibility, BLEST stops delivering data via that SF for some time. This implies that if the network path with the highest RTT is the only one accessible, BLEST can choose to wait for the lowest RTT path to become available again if it believes that transmitting on the highest RTT path would cause the receiver to be blocked. In basic form, it waits for the shortest RTT SF to become available, even if other SFs could have room in their CWND, to prevent HoL blocking at the receiver [18]. However, this strategy cannot produce the best results in all cases since it may result in considerable packet delivery delays, especially for larger files, when one path remains unused, resulting in a waste of resources [19]. Moreover, the real-time path characteristics changes are not considered while making the prediction or scheduling decision. As a result, it may often mislead BLEST to pause sending through the best available paths, especially for wireless links. Thus, BLEST also fails to be a generic scheduling solution for the MPTCP [17].

## 3. Low Latency and High Data Rate (LLHD) Scheduler

In this section, we discuss the proposed LLHD scheduler in detail. As mentioned earlier, for successfully avoiding HoL blocking at the receiver, the schedulers should be aware of the continuously changing environment of the network. The key network parameters include delay, path loss, and BW. We designed the LLHD in such a way that it keeps track of all these parameters in real-time and performs the scheduling by systematically considering these parameters.

The goodput of an SF represents how much of the actual data can be sent through that SF. Thus, it consists of both BW and path loss information, and can be calculated as follows [20]:

(1) Goodput=Throughput−Losses

Moreover, RTT represents the delay in data transmission. The SF that has the highest goodput and lowest RTT can be regarded as the best SF for data transmission. Now, to select the best SF for sending a data packet, the LLHD defines a utility function (*γ*) as follows:

(2) $$\gamma =GP_{N}+\beta \times \frac{1}{RTT_{N}}$$

where *β* is the balancing factor, *GP_N_* and *RTT_N_* are the normalized goodput and RTT, respectively, and are defined as follows:

(3) $$GP_{N}=\frac{GP}{GP_{\max}}$$

(4) $$RTT_{N}=\frac{RTT}{RTT_{\max}}$$

where *GP*, *GP_max_*, *RTT*, and *RTT_max_* are the goodput of SF_i_, maximum goodput among all the SFs, RTT of SF_i_, and maximum RTT among all the SFs, respectively. Note that SF_i_ is a member of the set containing all the SFs with available CWND. Finally, the SF_i_ is selected as the best SF to send a packet, which has the highest value of *γ*. Moreover, the LLHD makes this comparison each time it receives an ACK by any of its SFs. Algorithm 1 summarizes the LLHD algorithm. Backup SFs are SFs that are specifically designated as such by the sender and are only utilized if no normal SFs are available. An SF is unavailable if it is no longer useable, and an SF is temporarily unavailable if the sender is unable to send because the SF is now inaccessible but may become available once the connection is restored.
**Algorithm 1:** Low latency and high data rate (LLHD) Scheduling algorithm**Initialization:***best_SF = null**ɣ_max = 0**β = 0.001**RTT_max = 9999999**GP_SF_max = 9999* **Upon reception of ACK:****for all** subflow *i*
**do** **if**
*SF_i is backup* **then**
  **continue**

 **end if**
 **if**
*SF_i is unavailable* **then**
  **continue**

 **end if**
 **if**
*SF_i is temp_unavailable* **then**
  **continue**

 **end if**

 *ɣ*
*_curr = (GP_SF_i/GP_SF_max) + β × (RTT_max/RTT_SF_i)*
 **if**
*CWND_available_for_SF_i*
**and**
*ɣ_curr > ɣ_max* **then**
  *ɣ*
*_curr =* *ɣ*
*_max*

  *best_SF = SF_i*

 **end if**
**end for****return** *best_SF*

## 4. Performance Evaluation

This section evaluates the performance of the LLHD and the MPTCP schedulers available in the Linux Kernel with respect to the throughput and flow completion time (FCT). A Mininet [21] emulator was used for the performance comparison. We first describe the experimental setup, then compare the performance.

### 4.1. Experimental Setup

As mentioned earlier, the Mininet emulator was used for the experiments. We coded the LLHD scheduler for MPTCP Linux Kernel v0.93.4, compiled it with “make”, and installed it using “insmod”. The code for the LLHD can be found in the GitHub repository given in [22]. The emulation time was 300 s and the CCA was LIA [23].

**Figure 1:**
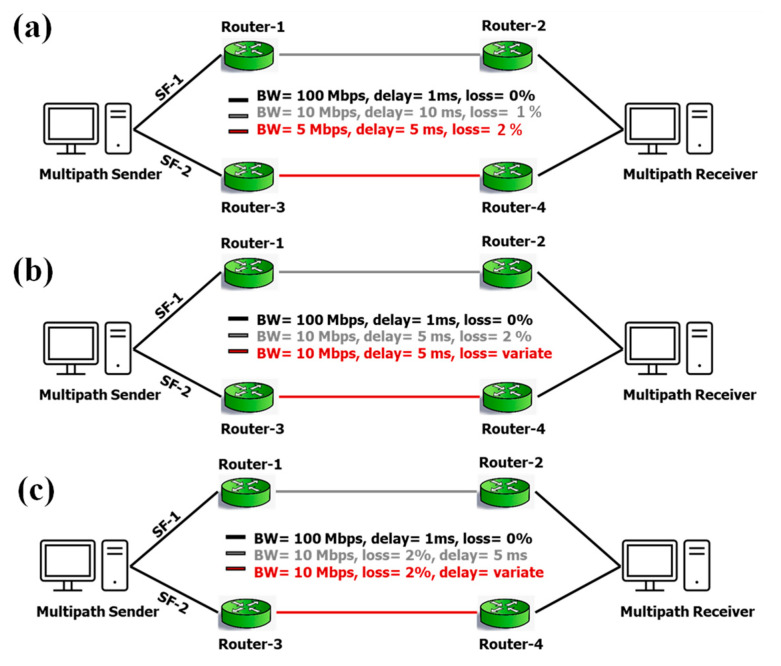
Scenarios for evaluating the considered schedulers (**a**) Scenario #1, (**b**) Scenario #2, and (**c**) Scenario #3.

We considered three emulation scenarios for the performance comparison, as shown in Figure 1. Scenario #1 explores two separate SFs linking the multipath sender with the multipath receiver. Two SFs have two bottlenecks with a capacity of BW 10 Mbps and 5 Mbps, path latency of 10 ms and 5 ms, and path loss rate of 1% and 2%, respectively. In this instance, the high BW route has the high RTT with the lowest packet loss rate. Hence, the optimal approach is the high BW path with high RTT, rather than the lowest RTT option. Thus, the schedulers confront the issue of determining the ideal route based on BW rather than merely RTT.

Scenario #2 covers two separate SFs that link the multipath sender and recipient. Two SFs have the same bottleneck capacity of BW 10 Mbps and a delay of 5 ms. However, the path loss rate of SF-1 is 2%, whereas that of SF-2 ranges from 2% to 10%. Both paths in this case have the same BW and RTT. However, the loss rate varies over time. Thus, rather than the RTT and BW, schedulers must pick the appropriate route in real-time based on the path with the lowest loss rate.

Finally, Scenario #3 has two separate SFs linking the sender and receiver. Two SFs have the same BW of 10 Mbps and a 2% loss rate. However, the delay for SF-1 is 5 ms, but the delay for SF-2 ranges from 5 ms to 25 ms. In this case, both pathways have the same BW and loss rate; nevertheless, the RTT varies. As a consequence, in this case, schedulers should choose the best route based on RTT rather than other factors, such as BW and packet loss rate.

### 4.2. Performance Comparison in Scenario #1

We begin by comparing the throughput and total data transmitted by the schedulers for Scenario #1. As seen in Table 1, the LLHD had the best throughput and can send the maximum amount of data. BLEST was the second-best performer due to its SF blocking system that avoids HoL blocking. The ECF was the next best performer, given its appropriate estimated arrival time for this scenario. It should be noted that the performances of all three schedulers were extremely close. On the contrary, SRTT and RR performed much worse. The lowest RTT route had low BW and packet loss rates, while the largest RTT path had high BW and low packet loss rates. As a result, the highest BW path is the optimum route for this scenario. The SRTT scheduler continued to transmit along the low RTT path while disregarding other criteria, such as packet loss rate and BW. As a result, it falls short of the LLHD, BLEST, and ECF. In the case of RR, the data were sent randomly among the SFs, yielding the worst performance. This result also shows that packet scheduling without a proper method cannot assure a significant performance for the schedulers. It should be noted that the CWND cap determined by the LIA is the reason why the throughput of all schedulers was relatively low compared to the overall capacity. Additionally, we will only provide the FCT results for the remaining scenarios since the throughput and FCT outcomes follow a similar trend.

**Figure 2:**
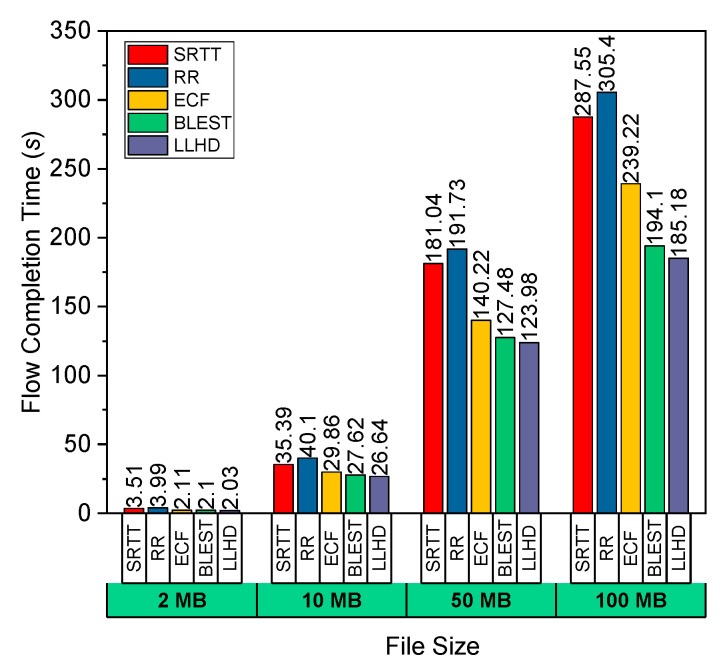
Performance evaluation of the considered schedulers in terms of FCT for variate file size in Scenario #1.

As shown in Figure 2, we conducted further tests in Scenario #1 by transmitting various data sizes and evaluating their FCT. We can see that the LLHD scheduler, with its smart route selection approach based on both the RTT and the goodput, achieved the lowest FCT for the various file sizes. As was previously indicated, by taking goodput into account, the LLHD not only analyzes the throughput per SF (i.e., the capacity of a route), but also takes path losses into account, since goodput excludes lost packets. The LLHD identified the subflow with the highest RTT as the best subflow in this scenario. It achieved optimum performance in terms of FCT and throughput by sending the most packets along the best route. BLEST was the second-best performer, owing to its built-in capacity to eliminate HoL blocking at the receiver. ECF comes in third place for performance. ECF sometimes can take more time than LLHD and BLEST. We argue that since there are random losses, and BLEST and ECF do not have the proper method to recognize the random link property changes and packet losses, they cannot make the best scheduling decision. This incorrect decision causes HoL blocking at the receiver, resulting in a prolonged FCT. As previously stated, the SRTT scheduler only took into account RTT and failed to accurately predict the network scenario in Scenario #1, resulting in an abnormally high FCT. Furthermore, RR performed the poorest since there was no suitable scheduling mechanism.

### 4.3. Performance Comparison in Scenario #2

**Figure 3:**
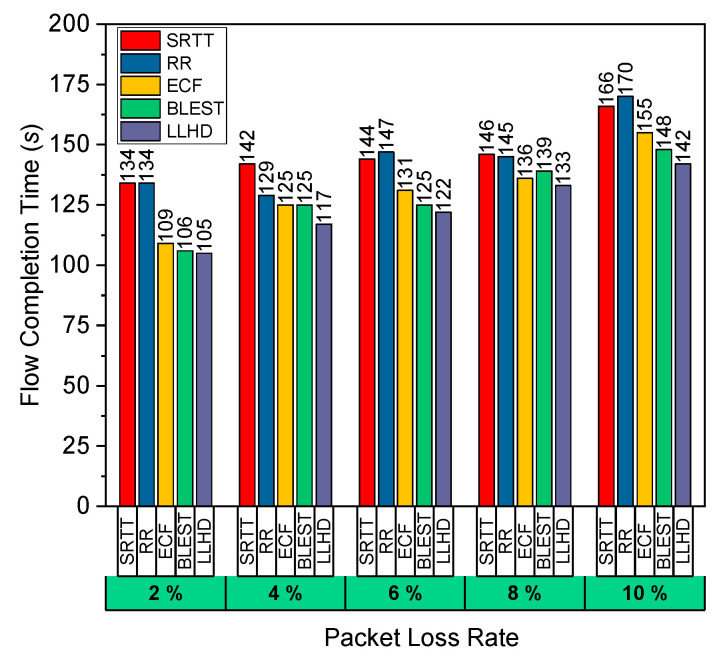
Performance evaluation of the considered schedulers in terms of FCT for variate packet loss rate in Scenario #2.

Using different packet loss rates throughout the path between Router-3 and Router-4, we examined the schedulers’ performance in Scenario 2. Both paths had the same latency and BW. Additionally, a 2% packet loss was specified for the link between Router-1 and Router-2. Again, due to its advanced algorithm, which considers packet losses while making scheduling decisions, the LLHD was able to achieve the lowest FCT in this trial, following the FCT results presented in Figure 3. RR, which distributes packets arbitrarily among the SFs, showed the poorest performance with the greatest FCT. After LLHD, BLEST, ECF, and SRTT exhibited the lowest FCT, sequentially, for the same reasons as previously indicated.

### 4.4. Performance Comparison in Scenario #3

**Figure 4:**
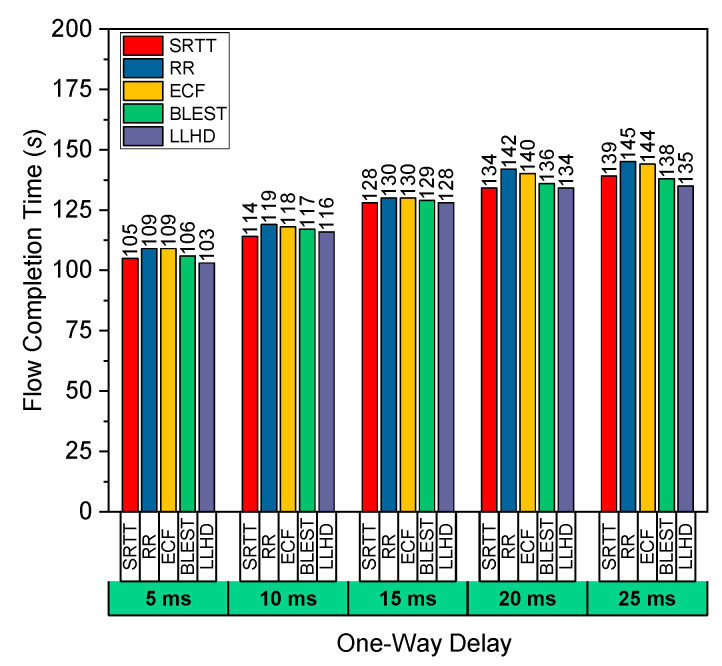
Performance evaluation of the considered schedulers in terms of FCT for variate path delay in Scenario #3.

Scenario #3 tested the schedulers by varying the delays in the path between Router-3 and Router-4. Other characteristics, such as BW and packet loss rate, were the same for both routes. We fixed the delay between Router-1 and Router-2 to 5 ms. As seen in Figure 4, the LLHD obtained the lowest FCT. However, in a 10 ms delay, the SRTT scheduler achieved the lowest FCT. In addition, it tied with the LLHD in the 15 ms and 20 ms delay situations and had very close FCT in the remaining cases. As previously mentioned, the SRTT scheduler prioritizes the shortest RTT path and attempts to route the majority of packets down that path. As a result, because the path between Router-1 and Router-2 clearly has the lowest RTT, the SRTT scheduler decided that the path between Router-1 and Router-2 was the best path and attempted to send the majority of packets through that path, achieving significantly better performance in this scenario. The LLHD yielded roughly the same, since it also chooses routes based on RTT. It also incorporates the available BW and loss rate in the scheduling choice by taking goodput into account. The performance of the remaining schedulers followed the same pattern as previously observed. The RR, on the other hand, performed the poorest owing to its lack of a solid scheduling mechanism and dependence only on randomness.

### 4.5. Performance Comparison in a Comprehensive Scenario

**Figure 5:**
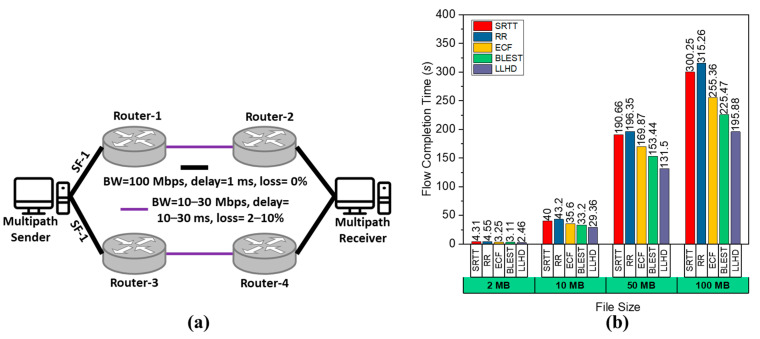
Performance evaluation of the considered schedulers in terms of FCT for a comprehensive scenario: (**a**) considered comprehensive scenario and (**b**) observed FCT.

Until now, we have observed the performance of the considered MPTCP schedulers in scenarios where one parameter was variable, and the others were fixed. To grasp the overall performance where all the parameters variate, as seen in the real-world scenarios, we performed experiments with the scenario shown in Figure 5a. The route connecting Router-1 to Router-2 and Router-3 to Router-4 has a variable BW ranging from 10 to 30 Mbps, a latency ranging from 10 to 30 ms, and a packet loss rate ranging from 2 to 10%. These factors change throughout time. We transmitted files of various sizes and executes 30 emulation runs for each file size. The average FCT is shown in Figure 5b. As we can see, the LLHD easily outperformed the others due to its smart scheduling decision-making, which takes into account changing path characteristics. The ECF and BLEST suffered due to their lack of path characteristics awareness, whereas the SRTT and RR performed the poorest, since they are entirely depending on RTT and randomness, respectively.

### 4.6. Performance Comparison in a Real-World Experiment

**Figure 6:**
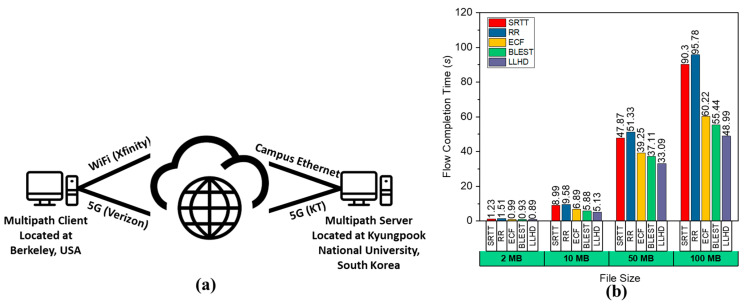
Performance evaluation of the considered schedulers in a real-world experiment: (**a**) real-world test setup and (**b**) observed FCT.

Finally, we tested the proposed LLHD and the considered MPTCP schedulers in a real-world test experiment, as shown in Figure 6a. We set up the server at Kyungpook National University on an Ubuntu machine with an Intel Core i7 9700k processor and 16 GB RAM. The server was linked to the Internet through the campus Ethernet and the KT 5G network. The client was in Berkeley, USA, with an Ubuntu computer powered by an AMD Ryzen 5500U CPU and 16 GB of RAM. The client was connected to the Internet via Xfinity’s WiFi service and Verizon’s 5G Internet service. The client downloaded files with different sizes, such as 2, 10, 50, and 100 MB. We repeated the test at least 30 times for each scheduler, and the results are shown in Figure 6b. In accordance with the emulation findings, the LLHD outperformed the other schedulers due to effective scheduling decisions that consider all available information. The BLEST and ECF were also close, but their gaps are due to their lack of knowledge about network changes. Because of a lack of adequate scheduling methods, the SRTT and RR failed to produce satisfactory results.

## 5. Conclusions

In this paper, we concentrated on designing a novel MPTCP scheduler that can make proper scheduling decisions while taking into account real-time network changes. We developed an LLHD scheduler that considers changes in network metrics, such as latency, path loss, and BW, and makes an appropriate scheduling choice based on the dynamic changes. We included the LLHD in the MPTCP Linux Kernel and made the source code publicly accessible. In extensive emulation studies, the LLHD effectively dealt with heterogeneous and constantly changing network characteristics, and efficiently used the available path capacity of the underlying network in contrast to current schedulers.

The downside is that our proposed method works reactively, attempting to effectively make the scheduling decision after loss occurrences or changes in the network. In a future study, we would want to integrate a method for predicting path characteristics changes, so that the LLHD may make scheduling decisions while taking projected network changes into account.

## Figures and Tables

**Table 1:** Throughput and total sent data for the considered schedulers.

Schedulers	Throughput (Mbps)	Total Sent Data (MB)
SRTT	2.17	79
RR	2.15	75
ECF	2.83	100
BLEST	2.84	101
LLHD	2.90	104
